# Machine learning models to predict post-dialysis blood pressure in children and young adults on maintenance hemodialysis

**DOI:** 10.1038/s41598-023-46171-3

**Published:** 2023-11-04

**Authors:** Raed Bou-Matar, Katherine M. Dell, Amy Bobrowski

**Affiliations:** Cleveland Clinic Children’s and Lerner College of Medicine of Case Western Reserve University, Cleveland, OH USA

**Keywords:** Renal replacement therapy, Computer science, Hypertension

## Abstract

Hypertension is associated with significant cardiovascular morbidity. Blood pressure (BP) control on maintenance hemodialysis (HD) is strongly impacted by volume status. The objective of this study was to assess whether machine learning (ML) is effective in predicting post-HD BP in children and young adults on HD. We collected data on BP, IDWG, pulse, and weights for patients on maintenance HD (> 3 months). Input features included DW, pre-post weight difference, IDWG and pre-HD BP. Seven models were trained and tuned using open-source libraries. Model performance was evaluated using time-series cross-validation on a rolling basis (3–12 month training, 1-day testing). Various regression scores were compared between models. Data for 35 patients (14,375 HD sessions) were analyzed. Extreme gradient boosting (XGB) and vector autoregression with exogenous regressors (VARX) achieved better accuracy in predicting post-dialysis systolic BP than K-nearest neighbor, support vector regression (SVR) with radial basis function kernel and random forest (p < 0.001 for each). The differences in accuracy between XGB, VARX, SVR with linear kernel, random forest and linear regression were not significant. Using clinical parameters, ML models may be useful in predicting post-HD BP, which may help guide DW adjustment and optimizing BP control for maintenance HD patients.

## Introduction

End stage kidney disease (ESKD) creates a high burden on families and the health system. Children and young adults with ESKD are at increased short and long-term risk of cardiovascular morbidity and mortality^1^. Such risk is, in part, attributed to the high prevalence of chronic uncontrolled hypertension and left ventricular hypertrophy (LVH)^2–4^. To optimize blood pressure (BP) control, patients receiving hemodialysis (HD) undergo frequent adjustment of fluid removal to achieve the “dry weight” (DW), defined as the lowest weight a patient on HD can tolerate while remaining normotensive and asymptomatic. Unfortunately, there is no accurate method to estimate DW of children or adults. Pediatric nephrologists rely on their experience and clinical judgment when reviewing trends in vital signs and other parameters, adjusting fluid removal targets up or down with each dialysis session, by trial and error, to approximate the true DW. Given the challenges and imprecision associated with such an approach, more reliable and efficient methods are crucially needed.

Machine learning (ML) is a type of artificial intelligence that allows computers to learn from data trends without being explicitly programmed. Prior attempts have been made to assess estimated DW, predict blood pressure trends, and predict the risk of hypotension using ML algorithms in the adult HD population^5, 6^.

Bi et al. proposed a time series-based regression model to predict DW in patients on HD^7^. They achieved remarkable accuracy (> 95%) in predicting DW within a 0.5 kg absolute error margin. More recently, Inoue et al. developed a random forest classifier model to help guide dry weight adjustment in hemodialysis patients^8^. On the other hand, the evidence for the utility of ML in children on HD is very limited. A pilot study from France reported a neural network ML approach to predict estimated DW in children using bioimpedance parameters, blood volume, and BP as inputs. They concluded that artificial intelligence has the potential to outperform experienced pediatric nephrologists in assessing DW in children on maintenance HD^9^.

Direct prediction of BP outside the HD setting is an area of active research. Su et al. proposed a novel multilayered Long Short-Term Memory (LSTM) network model that achieved remarkable accuracy for predicting long-term BP trends in healthy individuals using photoplethysmography and electrocardiogram data as input features^10^. Liu et al. also used photoplethysmography as a cuffless method to estimate BP with a mutli-dimension regression model^11^. Whether post-HD BP can be reliably predicted in patients on HD using ML models remains unknown.

In this study, we hypothesized that ML algorithms can predict post-dialysis systolic and diastolic BP of individual patients receiving maintenance HD using historical trends in pre-dialysis BP, DW, pre- and post-weights, heart rate, and intra-dialytic weight gain (IDWG) as input features.

## Methods

### Study design and population

The study was conducted with approval from the Cleveland Clinic Institution Review Board. Since the study involved analysis of existing clinical data obtained from the electronic health record, a waiver of consent was granted.

We retrospectively reviewed clinical parameters in patients with ESKD (aged 2 to 26 years) who received maintenance HD for at least 12 sessions from 2011 to 2021. We excluded patients who received HD only as an inpatient or received less than 3 months of outpatient HD treatment. A system to predict post-HD BP values was developed using various ML models to assist clinicians in determining optimal DW.

Patients received HD 3 days per week with most sessions lasting 3–4 h each.

### Inputs and output

Our data set included demographic data, DW, pre- and post-weights, pre- and post-BPs, pre- and post-heart rate values, and IDWG. *Input* features included patient-specific historical values of pre BP, IDWG, DW and weight loss (pre-weight − post-weight). BP was measured using oscillometric methods on certified devices according to the recommendations of the American Heart Association. Weights were obtained before and after dialysis using a calibrated scale. DW was the prescribed target weight entered by the healthcare provider into the electronic medical records for each HD session. This weight is generally defined as the lowest tolerated post-HD weight at which there are minimal signs or symptoms of hypovolemia or hypervolemia, as determined by the treating provider. The input sample size included HD sessions done on Mondays, Wednesdays and Fridays (3 times per week) for a minimum duration of 3 months (39 observations) and up to a maximum duration of 12 months (156 observations). *Output* included post-HD systolic and diastolic BP. We did not aim to predict the patient’s DW directly since there is no accepted "gold standard" for DW. Instead, clinicians are expected to utilize the BP predictions to make more informed decisions regarding the estimated DW.

### Data imputation, pre-processing, fitting, and cross validation

Data preprocessing included “robust scaling” in which the median was removed, and the data was scaled based on the interquartile range independently for each feature. This scaling was used to minimize the impact of outliers. No imputation was deemed necessary since sessions with missing values were excluded for models that cannot handle missing values, particularly support vector machines regression. Feature selection, hyperparameter tuning, and fitting were performed using open-source Scikit-learn^12^, XGB^13^, and Statsmodels^14^ Python libraries. To help improve the model's performance and scalability, input features were selected based on the initial descriptive analysis. It should be noted that the feature weights were adjusted using exponential decay (*y* = *e*^*x/*30^), to emphasize more recent observations, slightly improving the performance of the models.

Seven ML models were selected for evaluation based on feasibility, known utility in time series forecasting and overall speed of execution:

1. Support vector machines regression with linear kernel (SVRL)^15^
2. Support vector machines regression with radial basis function kernel (SVRR)^15^
3. K-nearest neighbor (KNN)^16^
4. Random forest (RF)^17^
5. Linear regression (LR)^12^
6. Extreme gradient boosting (XGB)^13^
7. Vector autoregression with exogenous regressors (VARX)^14^

Hyperparameters were tuned using time-series cross-validation to determine the best-performing models. The settings and optimized hyperparameters for each model are listed in Supplementary Table S1.

### Model evaluation and statistics

Baseline characteristics were reported as percentages for categorical variables. Mean and standard deviation were reported for normally distributed continuous, medians for continuous variables that are not normally distributed. Model performance was evaluated using time series cross validation on a rolling basis (3–12 months training, 1 day testing). Model performance on the training and testing folds were compared to avoid over-fitting. Statistical analysis was performed using Scikit-learn^12^, Statsmodels^14^, and Scipy^18^. The performance of regression ML models on training and testing folds was compared using a variety of scores including the Mean Absolute Percentage Error (MAPE), the r-squared score (*r*^2^), the root mean square error (RMSE) and the mean absolute error (MAE). The percentage of predictions that fell within 10% and 30% of the target values were also calculated. We chose the MAPE as the primary score for parameter tuning and model comparison. The MAPE is widely used in forecasting regression models due to its simplicity and being more intuitive in its interpretation as compared to the other scoring parameters. Pairwise comparisons between model MAPE values were analyzed using Nemenyi post-hoc test. A p-value < 0.05 was considered statistically significant.

### Time series statistical model development

Time series data used to train our models was collected over a relatively short duration (3–12 months) and confirmed to be stationary using the augmented Dickey-Fuller test (p < 0.001). The VARX model was used rather than conventional autoregressive integrated moving average (ARIMA) due to its ability to handle multiple time series and exogenous variables, which is not possible with the standard ARIMA models. The VARX model was utilized to predict two variables (systolic BP and diastolic BP) while enforcing stationarity of the time series data and concurrently addressing the underlying autocorrelation between proximate BP values. Based on analysis of autocorrelation function (ACF) and partial autocorrelation function (PACF) plots, first-order autoregression (p = 1) and zero-order moving average (q = 0) were selected. In this case, post-HD BP was predicted as a time series endogenous variable, while pre-HD BP, the weight difference (pre-HD vs. post-HD), the DW and the IDWG were included as exogenous parameters to optimize the forecast precision^14^.

## Results

A total of 35 patients (14,375 sessions) were included in the final model training and cross validation. The sample consisted of 64% females, mean age 16 ± 3.5 years. Average IDWG was 1.88 ± 1.6 Liters and net ultrafiltration rate was 9.15 ± 6.5 mL/kg/hour.

### Model comparison

**Figure 1:**
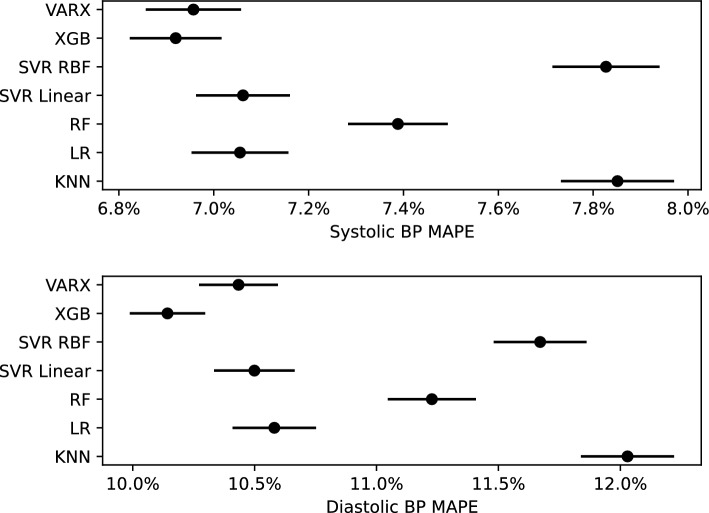
Plots comparing Mean Absolute Percentage Error (MAPE) values and confidence intervals of ML models used to predict post-hemodialysis systolic BP (Top) and diastolic BP (Bottom). *VARX* vector autoregression with exogenous regressors, *XGB* Extreme gradient boosting, *SVR* support vector machines regression, *RBF* radial basis function, *LR* linear regression, *RF* Random Forest, *KNN* K-nearest neighbor.

**Figure 2:**
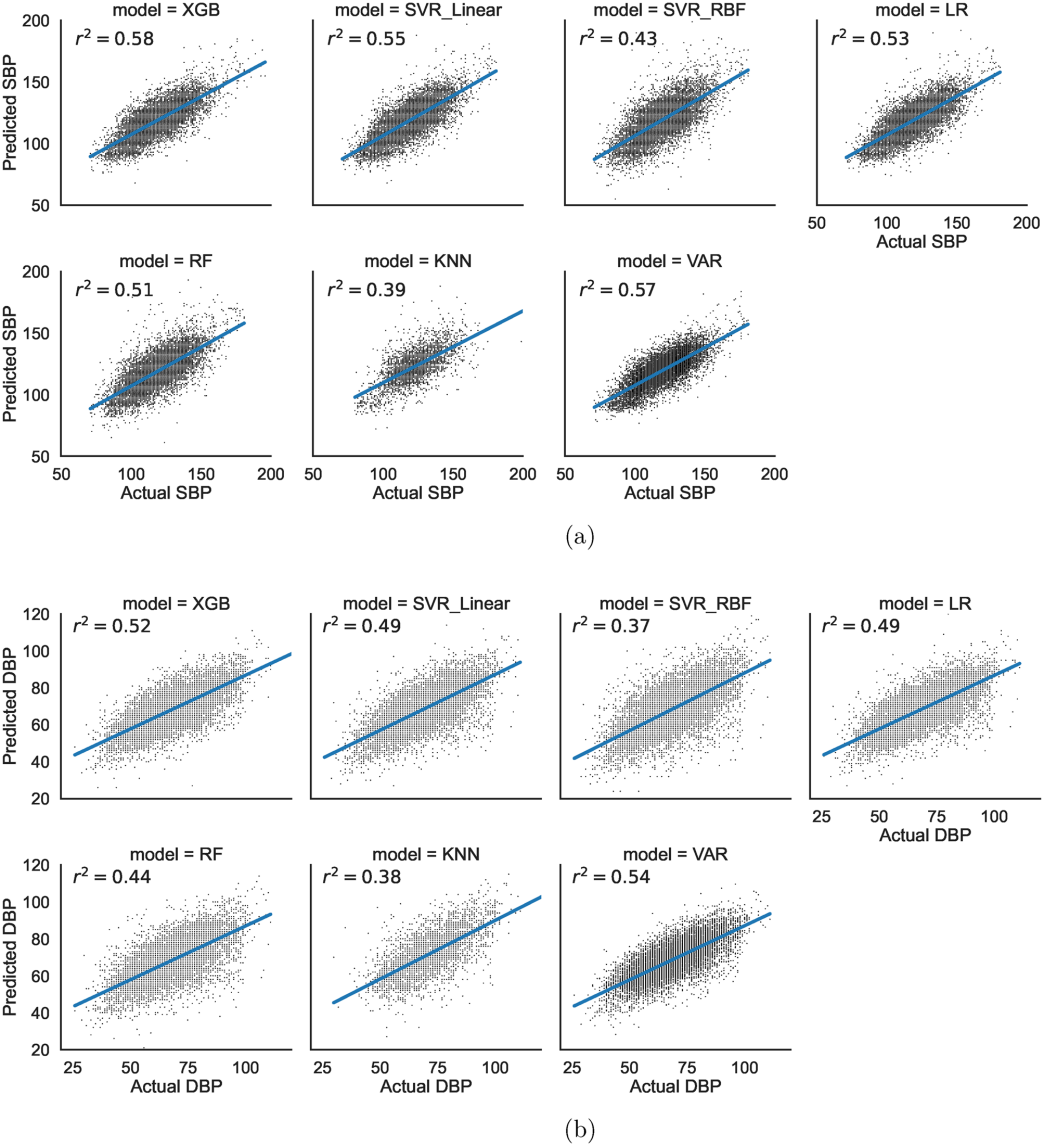
Scatter Plots of model predictions versus actual post-HD *systolic* BP values (**a**) and *diastolic* BP values (**b**); *r*^*2*^ r-squared coefficient of determination, *RF* Random Forest, *XGB* extreme gradient boosting, *LR* linear regression, *KNN* K-nearest neighbor, *RBF* radial basis function.

XGB was superior to RF (p < 0.001), KNN (p < 0.001) and SVRR (p < 0.001) in predicting systolic BP. Similarly, XGB was superior to RF (p < 0.001), KNN (p < 0.001) and SVRR (p < 0.001) in predicting diastolic BP. VARX also performed better than RF (p < 0.05), KNN (p < 0.001) and SVRR (p < 0.001) in predicting systolic and diastolic BP. The differences between XGB, VARX, SVRL and LR were not statistically significant (Fig. 1). Comprehensive regression scoring parameters for all models are listed in Table 1. Scatter plots of actual versus predicted systolic and diastolic BP values are shown in Fig. 2.

**Table 1:** Comparison of model evaluation parameters.

	r^2^	MAPE	RMSE	MAE	Within 10%	Within 30%
XGB						
Systolic BP	0.58	6.9%	10.6	8.2	76.2%	99.6%
Diastolic BP	0.52	10.1%	8.9	6.9	60.8%	96.3%
SVRL						
Systolic BP	0.55	7.1%	10.7	8.3	75.4%	99.5%
Diastolic BP	0.49	10.5%	9.3	7.0	59.6%	95.6%
SVRR						
Systolic BP	0.43	7.8%	11.9	9.2	71.4%	99.0%
Diastolic BP	0.37	11.7%	10.3	7.8	55.9%	93.9%
Linear regression						
Systolic BP	0.53	7.1%	10.7	8.3	75.6%	99.4%
Diastolic BP	0.49	10.6%	9.3	7.0	60.0%	95.3%
Random Forest						
Systolic BP	0.51	7.4%	11.2	8.7	73.4%	99.3%
Diastolic BP	0.44	11.2%	9.7	7.4	57.1%	94.4%
KNN						
Systolic BP	0.39	7.9%	12.1	9.2	71.0%	98.9%
Diastolic BP	0.38	12.0%	10.5	8.0	54.2%	93.2%
VARX						
Systolic BP	0.57	7.0%	10.4	8.1	75.7%	99.6%
Diastolic BP	0.54	10.4%	8.8	6.8	58.8%	96.1%

All models were evaluated on testing data.

*r*^*2*^ r-squared score, *MAPE* mean absolute percentage error, *RMSE* root mean square error, *MAE* mean absolute error.

### Feature importance

**Figure 3:**
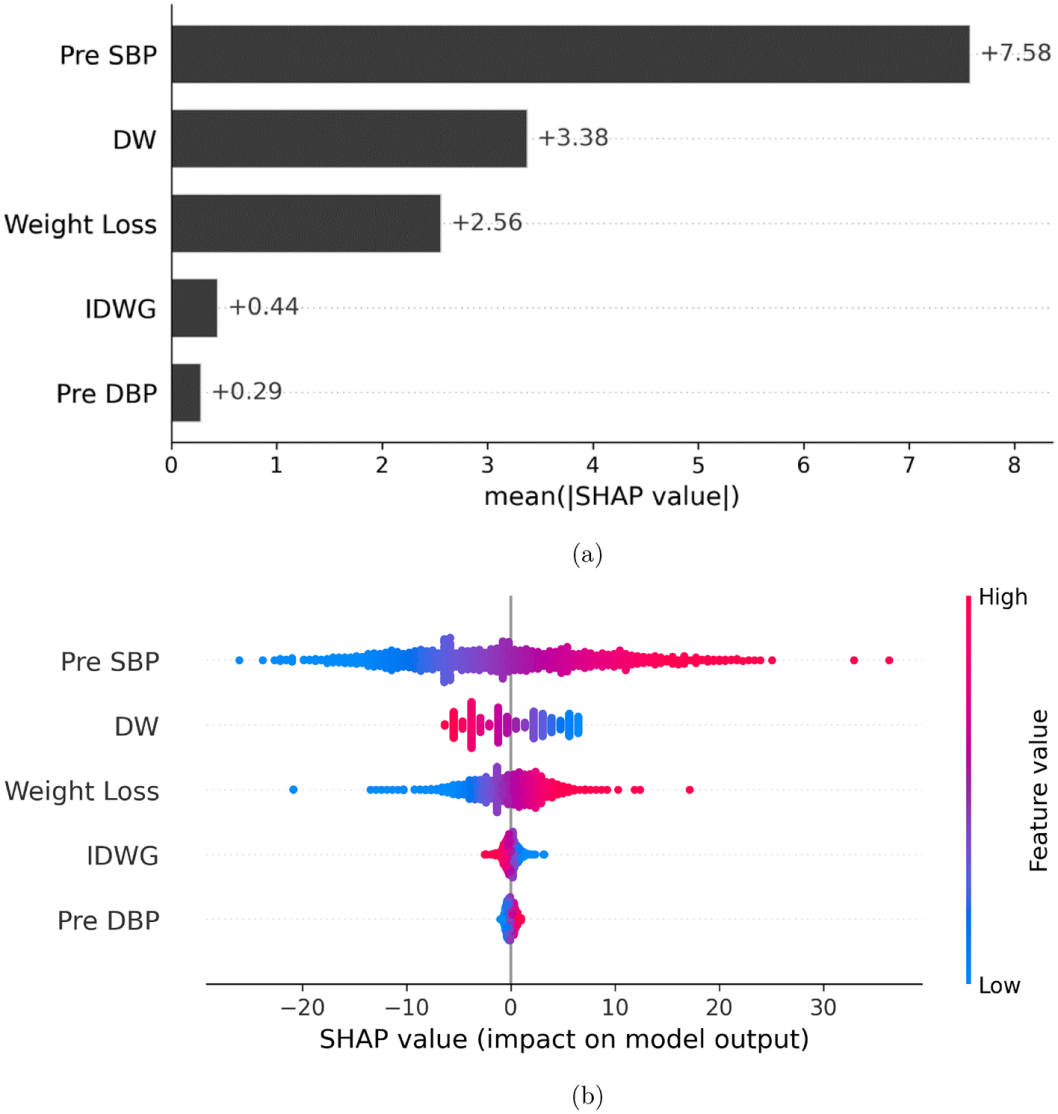
(**a**) Bar graph depicting XGB SHAP values for each feature of a randomly selected dialysis patient. (**b**) Beeswarm graph showing the distribution of SHAP values for each feature on XGB model predictions for a randomly selected dialysis patient.

SHapley Additive exPlanations (SHAP) values were utilized to help explain the output of the best performing model (XGB) as previously described by Lundberg et al.^19^. SHAP values are used to show the relative contribution of each feature based on game theory. In addition, SHAP values reveal the distribution of impact that each feature has on the general model predictions. Since the models were unique for each patient, variability was observed in the importance of features between models trained for different patients. However, overall trends emerged highlighting pre-systolic BP, pre-diastolic BP, IDWG and weight loss as the most important features. An example of relative feature importances as reflected by the SHAP values for a randomly selected patient using XGB model are depicted in Fig. 3.

## Discussion

In this proof-of-concept study, we developed and cross-validated a ML algorithm that predicts post-HD systolic and diastolic BP, using previous trends in BP, DW, pre-post weight difference and IDWG as input features. The predicted values can be used along with clinical judgment to determine the need to maintain, increase, or decrease the current estimated DW.

Our study included a representative sample of children and young adults over a 10-year period from a moderate sized pediatric dialysis unit. While a small percentage of records were excluded due to missing data, it is likely that the missing entries in the electronic medical records occurred at random and are unlikely to have biased our trained models. Our time-series cross validation approach allowed us to simulate real-life scenarios and closely estimate prospective performance of the models. The study evaluated and compared a variety of well-established robust ML algorithms that have been previously used in various healthcare, business, and technology applications.

XGB and VARX models were overall superior to other models in terms of overall accuracy. Since VARX is a statistical model, training was more computationally expensive, requiring an average of several minutes per patient on a regular office desktop computer. This renders such a model impractical for use in a real-time training and prediction scenario. In addition, VARX model was also more sensitive to outliers resulting in deterioration in model accuracy on datasets that contain a relatively large number of outliers. Such limitations could not have been predicted prior to starting the training and testing procedures and may vary by the specific computer system being used. XGB provided the best balance between accuracy and performance in our experience. This is not unexpected since XGB offers several advantages over other models, including: (1) its native ability to handle missing values, (2) built-in L1 (lasso regression) and L2 (ridge regression) regularization and (3) use of parallel processing^13^.

Previous data by Niel et al. used bioimpedance, blood volume monitoring, and BP as input features for neural networks to help determine the optimal DW. The authors concluded that the neural network models have the potential to outperform experienced nephrologists in optimizing dry weight^9^. They considered their neural network interface to be user-friendly and could be accessed directly from the Internet, on a smartphone or computer. They highlighted impediments to ML processes including need to retrain the neural network with any change in target population, need to ensure a friendly user-interface. Similarly, Bi et al. developed a time series-based regression model that predicted weight fluctuations in adult HD patients using Electronic Health Records (EHR)^7^. More recently, Inoue et al. took a different approach using a random forest classifier to predict the probability of adjusting DW at each dialysis session^8^. In this study, we chose to predict BP instead since there is currently no "gold standard" method to measure DW and therefore the accuracy of a ML model to predict DW cannot be reliably measured. We are not aware of any studies that have used readily available data, such as weight, BP and pulse trends as input features to predict BP post-HD. To our knowledge, this is the first study to investigate real-time predictions of post-HD BP in children and young adults on HD using ML approach. We hypothesize that such predictions may result in improved BP control in pediatric HD patients through improved estimation of the true DW and/or improved antihypertensive medication management.

Intra-dialytic blood volume monitoring (Crit-Line, Fresenius Renal Technologies, Waltham, MA) use real-time changes in blood hematocrit as a reflection of patient fluid status, thus help reduce the risk of intra-dialytic hypotension. Based on the Crit-line results, providers adjust fluid removal targets to achieve desired DW. Initial smaller studies promised that blood volume monitoring would be effective in preventing intra-dialytic morbidity^20, 21^. However, subsequent larger randomized controlled studies failed to confirm such benefit^22, 23^. Blood hematocrit changes generally correlate well with relative changes in fluid status but are likely not be as useful as ML in predicting post-HD BP.

The use of ML in clinical practice in general, and in dialysis, carries many advantages. ML models are relatively inexpensive and can help offset the burden of analyzing large volumes of data. A ML model may recognize patterns beyond what is feasible within the time constraints of a physician–patient interaction. It is conceivable that in the soon ML models would allow for accurate personalized predictions of dialysis outcomes for each patient. This will guide clinicians to make more informed adjustments of various dialysis parameters in real-time, enhancing overall safety of the dialysis procedure. For example, a dialysis provider may use our model to automatically predict post-HD BP at each outpatient encounter. If the predicted BP falls outside the target range, the provider may then make hypothetical changes to DW and run the model again to generate new predictions. Based on the repeated predictions, the provider uses her clinical judgement to decide on next steps in management (adjust DW, adjust BP medications or simply continue to monitor).

Our study has several limitations. The relatively small sample size, a common issue in most pediatric dialysis studies, may limit the generalizability of our results. While our models’ accuracy scores were overall reasonable based on the MAPE and percentage of predictions that fall within 10% and 30% of the measured BP, the r-squared scores were relatively low. This is likely due to the multitude of other factors that influence BP in HD patients and are more difficult to measure (e.g. emotional state and changes in diet). Note that excessive IDWG has been associated with higher salt intake in-between dialysis sessions so this variable may in part capture changes in the diet of this specific patient population^24^. The input features in this study were chosen because they were practical, routinely measured in day-to-day clinical care and because health care providers commonly use such trends to adjust the HD prescription and/or antihypertensive medications. Furthermore, while the long-term potential of an ML approach is evident, advancement to a clinical practice application requires further study. The model performance will need to be tested on hard outcomes in a controlled study design. Finally, correlation of model predictions with 24-h ambulatory BP monitoring (ABPM) would have been helpful. ABPM is known to better correlate with longitudinal cardiovascular outcomes^25^. However, due to their simplicity, frequency and widespread availability, post-dialysis BP measurements are still commonly utilized in day-to-day practice for the purpose of adjusting DW and antihypertensive therapy. ABPM on HD patients is generally performed in most centers once or twice per year to confirm adequate long-term BP control and is not considered practical for daily or weekly monitoring.

In conclusion, utilizing vital signs trends and other readily available parameters, ML models may prove useful in predicting post-HD BP in children and young adults with ESKD. Such predictions are intended to guide DW adjustment and antihypertensive therapy, supplementing clinical judgment.

### Supplementary Information

Supplementary Table S1.

## Data Availability

The datasets generated and/or analysed during the current study are not publicly available due to protect the privacy of the individuals included in the study but are available from the corresponding author on reasonable request.
