# Supplementary material for: Machine learning models to predict post-dialysis blood pressure in children and young adults on maintenance hemodialysis
Source: Sci Rep. 2023 Nov 4;13:19105. doi: 10.1038/s41598-023-46171-3 (PMC10625550; doi:10.1038/s41598-023-46171-3)
Supplement: Supplementary file 1 — Supplementary Table S1. [file 41598_2023_46171_MOESM1_ESM.pdf]

Machine Learning Models to Predict Post-dialysis Blood Pressure in Children and Young Adults on Maintenance Hemodialysis

Raed Bou-Matar*, Katherine M. Dell, and Amy Bobrowski

Cleveland Clinic Children's and Lerner College of Medicine of

Case Western Reserve University, Cleveland, Ohio, United States

* *Corresponding Author*: Raed Bou-Matar (boumatr@ccf.org)

Supplementary Table S1: Model Settings and Optimized Tuning Parameters

**Computer system**	HP EliteDesk 800 G6 Small Form Factor PC<br>Intel(R) Core i5-10500 CPU @ 3.10GHz<br>3096 Mhz, 6 Core(s), 8 GB RAM
**Python libraries**	Pandas 2.0<br>Numpy 1.24.2<br>Scikit-learn 1.2.2<br>XGBoost 1.7.5<br>Statsmodels 0.13.5
**Extreme gradient boosting**	booster = gblinear<br>eta = 0.5<br>gamma = 0<br>max_depth = 6<br>min_child_weight = 1<br>max_delta_step = 0<br>subsample = 1<br>sampling_method = uniform<br>lambda = 0.1<br>alpha = 0<br>tree_method = auto<br>random_state = 0
**SVR Linear**	epsilon = 2<br>gamma = 'scale'<br>tol = 1e-3<br>C = 0.6<br>max_iter = no limit
**SVR RBF**	epsilon = 2<br>gamma = 'scale'<br>tol = 1e-3<br>C = 0.6<br>max_iter = no limit
**Random Forest**	n_estimators = 400<br>criterion = "squared_error"<br>max_depth = None<br>min_samples_split = 10<br>min_samples_leaf = 1<br>min_weight_fraction_leaf = 0<br>max_features = 1<br>max_leaf_nodes = None<br>min_impurity_decrease = 0<br>bootstrap = True

**K-Nearest Neighbor**	n_neighbors = 20<br>weights = ‘uniform’<br>algorithm = ‘auto’<br>leaf_size = 1<br>p = 1<br>metric = ’minkowski’
**Linear Regression**	Not applicable
**VARX**	max_train = 120<br>p-order = 1<br>q-order = 0

Supplementary Table S1. System specifications and hyperparameter tuning parameters are listed for each trained model. VARX: Vector autoregression with exogenous regressors; SVR: Support vector machines regression; RBF: Radial Basis Function.
